# The SphKs/S1P/S1PR1 axis in immunity and cancer: more ore to be mined

**DOI:** 10.1186/s12957-016-0884-7

**Published:** 2016-04-29

**Authors:** Lei Jin, Wei-Ren Liu, Meng-Xin Tian, Jia Fan, Ying-Hong Shi

**Affiliations:** Department of Liver Surgery, Liver Cancer Institute, Zhongshan Hospital, Fudan University; Key Laboratory of Carcinogenesis and Cancer Invasion of Ministry of Education, 180 FengLin Road, Shanghai, 200032 China; Institutes of Biomedical Sciences, Fudan University, Shanghai, People’s Republic of China

**Keywords:** SphKs/S1P/S1PR1, Immunity, Cancer

## Abstract

Over the past two decades, huge amounts of research were launched to understand the functions of sphingosine. Many pathways were uncovered that convey the relative functions of biomacromolecules. In this review, we discuss the recent advances of the role of the SphKs/S1P/S1PR1 axis in immunity and cancer. Finally, we investigate the therapeutic potential of new drugs that target S1P signaling in cancer therapy.

## Background

Sphingosine was first described by J. L. in the 1880s, and its structure was characterized as 2S,3R,4E-2-aminooctadec-4-ene-1,3-diol [1]. In the last two decades, research has illustrated the significant functions of sphingosine in physiology, pathology, and the chemical or structural biology of metabolism.

Sphingosine kinases (SphKs) are comprised of two forms, SphK1 and SphK2, which are upstream of S1P. They catalyze the phosphorylation of sphingosine to S1P, regulating the amount of S1P [2]. The functions of these two S1P metabolic enzymes are critical. Double-knockout animals were embryonic lethal, due to the incomplete maturation of the vascular system and brain, although mice deficient in either SphK1 or SphK2 had no obvious abnormalities [3]. The differences in SphKs existed at an early stage. On a macro level, SphKs are activated by numerous stimuli, including chemokines, intercellular adhesion molecules, and pro-inflammatory cytokines [4, 5]. On a molecular level, SphK1-S225A, Calcium and Integrin-binding protein 1, Elongation Factor 1A (eEF1a), and hypoxia regulate the specific microenvironment of SphKs [6–8]. Sphingosine, SphKs, and S1P regulation was more complex in cell nuclei. Hait N. C. et al. reported that the S1P produced by SphK2 inhibited histone deacetylases (HDACs), which modulated the dynamic balance of histone acetylation and influenced the epigenetic regulation of specific target genes [9]. SphK2 inhibited cell growth and promoted apoptosis [2]. In addition, the binding of S1P was also required for the E3 ubiquitin ligase activity of TRAF2, an essential mediator of the nuclear factor-κB (NF-κB) pathway initiated by the major inflammatory signaling molecule TNF-α [10–12]. S1P may promote cancer progression via HDAC1/2 and NF-κB intracellular targets [4, 9, 10]. SphK1 was reported to contribute to S1P synthesis by a combination of epigenetic, transcriptional, and posttranslational mechanisms [13]. Therefore, SphK1 may have a more positive effect on S1P than SphK2.

It was unclear whether a SphKs/S1P regulatory loop existed in cells. Pappu R found that in an “S1P-less” mouse model, SphK1 and SphK2 were also disrupted in blood stem cells, vasculature, liver, and additional tissues [14].

Sphingosine-1-phosphate (S1P) is a common, complex molecule biosynthesized in our bodies. Its complicated structure forms via several steps. Sphingolipid biosynthesis is initiated in the endoplasmic reticulum (ER) [15]. Sphingolipid and S1P maintain a dynamic balance via a series of biosynthetic or recycling/degradation pathways and are phosphorylated or dephosphorylated by specific and nonspecific lipid phosphatases [2].

In the ER, sphingosine-1-phosphate phosphatase 1 (SPP1) and sphingosine-1-phosphate phosphatase 2 (SPP2), two sphingoid base-specific phosphatases, regulate S1P levels [16]. SPP1 gains access to extracellular S1P via its import into the cell by an ATP-binding cassette (ABC) transporter [17], similar to agonists which activate SphK1 by inducing its recruitment to the plasma membrane and producing S1P by “inside-out signaling” [18–21]. SPP2 influences branching morphogenesis during kidney development by influencing S1P levels in the metanephric mesenchyme [16].

As discussed above, SphKs hold important guide roles in this process. Degradation is also important to balance the amount of S1P. S1P is degraded by the intracellular protein S1P lyase. In this process, the sphingoid base is cleaved at position C2,3, yielding hexadecenal and ethanolamine phosphate [22]. S1P lyase was upregulated in response to ischemia, radiation, and chemical injury in other tissues [2] and was also regulated by PDGF signaling [23]. Similar to SphKs, S1P lyase also maintained a dynamic balance.

Interestingly, the S1P lyase level in the blood was much lower than that in tissues, leading to higher S1P levels in the blood and lower amounts in tissues. Erythrocytes and platelets lack S1P lyase or S1P phosphatase activity when they mature [14]. Hagen found that loss of S1P lyase (SPL) resulted in high, cytotoxic S1P levels in the neurons and vital organs [24]. This observation is contradictory to early studies in SPL null mice, which form pathological lesions in the lung, heart, urinary tract, and bone due to lack of S1P [9, 25]. Upstream of S1P, many molecules need further study.

The S1P receptors are regulated through distinct mechanisms. Transcriptional regulation of S1PR1 is controlled by Krüppel-like factor 2 (KLF-2), and S1PR1 is exquisitely sensitive because of ligand-induced internalization [26]. Although the S1P receptors emit signals in the presence of S1P, they also signal without S1P due to the downstream signaling of G protein partners [27]. Recent studies exposed an interesting phenomenon, in which Dynamin-2 was essential for S1PR1 internalization and promoted egress from both the thymus and lymph nodes wherever high S1P concentrations existed [28].

S1PR1 correlated with S1PR1-JAK/STAT3 signaling in Treg cells [29]. Its specific role in immunity is discussed below.

## The role of the SphK/S1P/S1PR1 axis in immune regulation

SphK1-generated S1P participates in many physiological and pathological processes. S1P binds to five-cell surface receptors, called S1PR1-5. S1PRs performed important functions. S1PR1 is particularly distinctive. It plays an important role in the mature vascular system, pathological angiogenesis, immune cell egress from tissue compartments, hematopoietic, vascular and stem cell survival, cytokine production, and so on [30–34]. An interesting study noted that SphKs-S1P exist in the mitochondria, where the balance of their amounts influences respiration [12].

Hematopoietic stem and progenitor cells (HSPCs/HSC/HPC) express S1P receptors. Following different S1P gradients, HSPCs emigrated from peripheral tissues to the lymphatic system [35, 36]. Coincidentally, the egress of B cell progenitors from the bone marrow (BM) depended on S1P/S1PR1 interactions [37]. Furthermore, S1PR1 was required for the steady-state trafficking of HSPCs. Suppression of S1PR1 expression by genetic or pharmacologic methods significantly impaired the mobilization of HSPCs into the blood. CXCR4 blockade or the S1PR1 agonist SEW2871 enhanced AMD3100-mediated HSC mobilization [38]. In addition, the release of S1P from mast cells and erythrocytes in the peripheral circulation was dependent on ATP-binding cassette (ABC) transporters. This phenomenon was similar in either physiological or pathological situations [21, 39]. Interestingly, Henrik Fyrst raised the critical view why, with the help of S1P receptors for immune cell egress, some specific functions of non-lymphoid immune cells existed, perhaps by an as yet unknown feedback mechanism from other cells that influence S1P-S1PR (especially S1PR1) sensitivity [2].

Apart from the function for HSPCs, S1P and its receptors influence many organs through systemic and localized chemotactic gradients [40, 41]. Studies investigated the mechanisms underlying these phenomena. In an inflamed lymphoid organ, type 1 interferons (IFNs) increased the lymphocyte expression of the activation antigen CD69, which bound S1PR1 and induced its internalization and degradation through the loss of CCR7 in newly generated effector T cells, where S1PR1 was upregulated [42, 43]. In the spleen, the S1P-S1PR1 axis changed cyclically, which allowed B cell migration in a CXC-chemokine receptor 5 (CXCR5-dependent manner toward CXC-chemokine ligand 13 (CXCL13), which is produced by follicular DCs [44]. Faroudi, M explained that S1PR1 played a crucial role in the reverse transmigration of lymphocytes by regulating the small GTPase RAC and potent actin nucleation, which were required for the reorganization of the actin cytoskeleton [45]. Allende, M. L. et al. [37], using B cell-specific S1PR1 knockout *(B-S1PR1*KO) mice, found that S1PR1 provided a signal necessary for the efficient transfer of newly generated immature B cells from the bone marrow to the blood.

Many viewpoints illustrate the phenomenon that lymphocytes internalize in the lymphoid organs is via the CCL21–CCR7 axis [42, 46, 47]. Recent studies illustrated an interesting phenomenon. Collagen expression was negatively regulated by S1PR1 and S1PR3 in human bone marrow-derived mesenchymal stem cells (hMSCs) [48]. The new focus may well be on collagen. One study found that S1PR1 and integrin ß4 (ITGB4) were essential for hepatocyte growth factor (HGF)-mediated EC barrier enhancement [49]. In brief, S1P and its receptors function on the macro-environment and microenvironment in the body via an “inside-out signaling” mechanism [4]. These cumulative findings proved that S1P is a major regulator of innate and adaptive immunity. At the same time, more questions arose, including whether S1P signaling contributes to inborn or acquired immune diseases, and its long-term effects on thymic education and peripheral lymphoid organ functions.

## The role of the SphK/S1P/S1PR1 axis in pathophysiology/pathogenesis

The SphKs/S1P/S1PRs axis regulates many physiological processes, including pathogenesis or pathophysiology. S1P and its receptors regulate allergic responses, lymphocyte differentiation, endothelial barrier integrity and cytokine and adhesion molecule expression, asthma, rheumatoid arthritis, sepsis, and inflammatory bowel disease [13, 18, 50–53]. IgE receptors on mast cells upregulated SphK1 (and probably SphK2), leading to S1P production. S1P in turn activated its own receptors in an autocrine and/or paracrine manner, which promoted mast cell activation and degranulation [54]. Inhalation of SphK inhibitors improved disease severity in a mouse model of asthma [55]. Histamine released from mast cells stimulated SphK1 and enhanced S1P production by both hematopoietic and nonhematopoietic sources, which was crucial for the clearance of histamine [33]. Camerer, E explained that S1P continuously activated luminal endothelial S1PR1 to maintain tight cell–cell junctions. Following the entry of S1P into the subendothelial space via “leaky” endothelium, dynamic S1PR1 signaling activated abluminal surface S1PRs to close intercellular gaps [50]. Endothelial cell S1PR1 maintained the homeostatic barrier property of the vascular system, and the SphK1/S1P/S1PR1 axis was involved in the restoration of normal vascular barrier integrity during infection and inflammation [50, 56].

In response to TNF and other cytokines, SphK1 was activated and translocated to the plasma membrane to catalyze the production of S1P, which was then exported out of the cell by specific transporters to activate its receptors in an autocrine manner. In addition, S1P activated the key inflammatory transcription factor nuclear factor-κB (NF-κB), which was found to be independent of S1PRs [57–59]. In sepsis, both TLR2 and TLR4 stimulated and upregulated SphK1 and downstream factors such as NF-κB. Therefore, deletion or inhibition of SphK1 prevented sepsis in mouse models of LPS challenge or cecal ligation and puncture [51]. This paved the way for the exploration of SphK1 inhibitors for the treatment of sepsis in humans [11]. In the liver, fibrosis is the common response to chronic liver injury, characterized by excessive deposition of collagen and other components of the extracellular matrix [60, 61]. As illustrated above, S1P played a critical role in lymphocyte egress from secondary lymphoid tissues and the thymus. Li C established acute and/or chronic liver injury by carbon tetrachloride injection or bile duct ligation (BDL) in mice, and found that S1P levels in liver tissue and serum were significantly increased [62]. S1P levels in the human fibrotic liver were increased through up-regulation of sphingosine kinase (SphK), irrespective of the etiology of fibrosis that led to liver fibrosis and cirrhosis.

SphK2 may also contribute to mast cell functions [63]. Lai, W.Q found that prior to the development of the clinical symptoms of arthritis, depletion of SphK2 enhanced the production of the pro-inflammatory cytokines IL-6, TNF, and IFNγ [64]. SphK2 inhibition alleviated disorders associated with immunosuppression, such as chronic infections and/or cancers [65]. Haberland, M [66] and Glauben, R [67] explained that S1P was bound to and inhibited histone deacetylases 1 (HDAC1) and HDAC2, which may be associated with human diseases such as cancer and inflammation. Furthermore, in Alzheimer’s disease, the activity of β-site APP-cleaving enzyme-1 (BACE1), the rate-limiting enzyme for amyloid-β peptide (Aβ) production, was modulated by S1P in mouse neurons. Both SphK inhibitors and overexpression of S1P-degrading enzymes decreased BACE1 activity, which reduced Aβ production [68]. Cardiomyocyte ischemia/reperfusion (I/R) injury is fatal. S1PR1 activation and downstream signaling through Akt enhanced ischemic preconditioning and mammalian cardiomyocyte survival [69]. At the same time, recent studies suggested that S1P agonists FTY720 /SEW2871 could provide protection against stroke and ischemic injury in many tissues [70]. In some studies, SPL inhibitors as well as S1P analogs mediated cardioprotection and prevented sepsis-related tissue injury. High-density lipoprotein (HDL) confers protection against atherosclerosis and heart disease. Recent studies found that S1P mediated HDL’s circulation through plasma carriers via its receptors [71]. S1P receptor agonists or antagonists may function in vasodilation, cardioprotection, and survival.

Unlike its positive role in cardiovascular disease, S1P signaling also contributes to carcinogenesis [72]. SphK1 is often upregulated in cancerous tissue, and its overexpression correlates with chemo- and radiation-resistance and poor prognosis [13]. SphK1 promoted cancer growth via multiple mechanisms, including S1P signaling to cancer cells. Sequestering S1P with a specific S1P monoclonal antibody blocked tumorigenesis and tumor angiogenesis in murine xenograft and allograft cancer models [73]. Based on these observations, inhibition of SphK1 reduced tumor growth, angiogenesis and chemoresistance in numerous xenograft models [72, 74].

## The role of the SphK/S1P/S1PR1 axis in cancer

Tumor cells secrete S1P, promoting growth, survival, motility, and metastasis via S1PRs in an autocrine/paracrine manner [75–77]. The specific processes are complex, including endothelial adhesion, angiogenesis, and regulation of tumor-stromal interactions [77] (see Table 1).

**Table 1:** SphK/S1P/S1PR1 axis in cancer

Which type	Main cancer/bad outcomes	Mechanism/causes	References
Sphk1↑	Breast cancer	Hemangiogenesis	[76, 77, 81, 85]
	Glioma	Lymphangiogenesis	
	B17 melanoma malignance	IL-17↑ → IL-6–Stat3↑ → stat3↑	
	MB49 bladder malignance		
SphK2 ↓	Colitis-associated tumorigenesis	SphK1↑	[80]
		SphK1/S1P/S1PR1 axis↑	
		NF-κB/STAT3 ↑	
S1P↑	Colon cancer	S1P lyase↓ → p53-, p38-dependent pathways ↑	[78, 79]
	Melanoma	SGPL1 gene ↑ → S1P lyase disruption ↑ → Bcl-2, Bcl-xL dependent pathways	
	Resistance to chemotherapy		
Stat3↑	Melanoma	RelA acetylation → NF-κB↑	[86, 87, 89]
	Myeloid cell-dependent tumor	BRAF–MAPK↑	
	A2058 tumor cells malignance	VEGF/bFGF → tube formation	

Interestingly, SPL and S1P phosphatase (SPP) expression were balanced in many human cancers and murine cancer models. In knockout mouse models of human SGPL1, which encodes SPL [78], and through quantitative real-time PCR (Q-PCR) and immunohistochemical analysis, studies found that SPL promoted apoptosis through p53- and p38-dependent pathways. SGPL1 gene which encodes SPL disruption in mouse embryonic fibroblasts increased their resistance to chemotherapeutic agents by upregulating Bcl-2 and Bcl-xL [79]. In some cancers, activated SPL affected carcinogenesis to cancer itself under stress [78]. Increasing S1P catabolism or inhibiting S1P biosynthesis could become a new way to treat cancer. In contrast, some studies found that the inhibition of S1P raised secondary malignancy [13]. Targeting S1P signaling may be a double-edged sword.

Studies found that signal transducer and activator of transcription-3 (STAT3) was important in the persistent activation of tumors [80, 81]. STAT3 is a transcription factor for S1PR1. Enhanced S1PR1 upregulated the expression of IL-6, a pro-inflammatory cytokine crucial for STAT3 activation, inflammatory cell-mediated transformation, and tumor progression [81]. The altered microenvironment is an important factor for malignant progression and metastasis [82].

Aberrant IL-6–JAK-STAT3 signaling in cancer cells is a major mechanism for cancer initiation, development, and progression [82–84]. Lee H used B16 cells, which have relatively low STAT3 activity and IL-6 expression in cell culture but greatly enhanced STAT3 activity in vivo, achieved at least in part through IL-6 production by the tumor stromal immune cells [85, 86]. Kujawski, M. et al., studied the role of STAT3 in tumor-associated MDSCs and tumor angiogenesis through C57BL/6 naive mice [87]. Chemotactic factors, including IL-6 and IL-10, correlated with STAT3, influencing the whole immune system, including the formation of metastatic niches [88, 89]. Studies have found minute differences in Jak somatotypes for STAT3, and various animal models were used to reveal the particular passageways in the microenvironment [29, 88, 90].

Several seminal studies have documented the importance of myeloid cells in providing a sanctuary for tumor cell adhesion, survival, and secondary site colonization [91, 92]. Myeloid cells mobilized and produced chemokines and other molecules in response to the tumor environment, thereby promoting cancer progression [93, 94]. However, the role of myeloid cells in forming a sanctuary for tumor cells in distant organs prior to tumor cell arrival/outgrowth remains unknown [91, 92]. Targeting STAT3/S1PR1 signaling in immune cells could reduce STAT3 activity and myeloid cell infiltration in future metastatic sites [91, 95, 96]. S1PR1-STAT3 in tumor cells and myeloid cells orchestrated premetastatic niche formation, and persistent STAT3 signaling in myeloid cells could increase their proliferation and survival, as well as that of other stromal cells at future metastatic sites [88].

## Targeting therapies toward the SphK/S1P/S1PR1 axis

In many types of cancer and in some inflammatory disease, SphK1 is overexpressed [18]. SphK1 expression, enzymatic activity, and subsequent S1P production were stimulated by TNF-α, as well as by a host of other inflammatory signaling molecules, including IL-1β, IFN-γ, IgE, and C5a [97]. SphK1 is accordingly a primary target for the development of therapeutic intervention strategies [98]. Safingol, a first-generation SphK inhibitor, had some achievements in phase 1 clinical trials [99] (see Table 2).

**Table 2:** Different drugs produce varied function in cancer

Drugs	Type	Main function	Functional mechanism	References
SK1-I	Inhibitor of SphKs	Leukemia	Bcl-2	[77, 100, 103]
		Breast cancer	ERK1/2 and Akt	
		Prostate cancer	MCF-7/MCF-7 HER2	
SKI-II	Inhibitor of SphKs	Human non-small cell lung cancer	PI3K/Akt/ NF-κB	[101, 102]
			Estrogen receptor-regulated genes	
		Breast cancer therapy	SDF-1	
Safingol	Putative inhibitor of SphK	Glioblastomas	A Phase I Clinical medicine	[99]
		Colorectal tumor		
		Adrenal cortical carcinoma		
		Sarcoma		
ABC294640	Selective inhibitor of SphK1	Breast cancer	Inhibition of tumor cell migration	[98, 106, 107]
		Ovarian cancer	Loss of microfilaments	
FTY720	Sphingosine analog	Peripheral blood stem cell (PBSC) transplantation	Internalization and degradation of S1P receptor 1–5	[35, 38, 40, 108, 109]
	Immunosuppressive small molecule drug	Anti-tumor in many tissues	Bone mineral metabolism	
			Enhanced AMD3100-mediated HSC mobilization	
		Needs further research		
		Relieved ovariectomy-induced osteoporosis	Prevent T and B cell egress from lymph nodes	
			S1PR1↑ → SDF-1 sensitivity↑ → CXCR4↑	

SK1-I,(2R,3S,4E)-N-methyl-5-(4′-pentylphenyl)-2-aminopent-4-ene-1,3-diol, designated SK1-I (BML-258), markedly reduced the growth of AML xenograft tumors and acute myelogenous leukemia by affecting multiple survival signaling pathways [100]. Kapitonov found that SK1-I reduced the growth, migration, and invasion of several glioblastoma multiforme (GBM) cell lines by using SphK1 inhibition. This function of SK1-I was attributed to the suppression of Akt activation, and the subsequent interruption of signaling through the Akt pathway, which is upregulated in the majority of glioblastomas [99]. Meantime, a combination of SphK1 inhibitors and cisplatin could be used to treat patients and provide some degree of disease regression [99].

SKI-II enhanced the resistance to apoptosis in human non-small cell lung cancer via the PI3K/Akt/NF-κB pathway [101]. Interestingly, SKI-II seemed to be relevant to hormone-associated diseases. SKI-II dose-dependently decreased estrogen-stimulated estrogen response element transcriptional activity and diminished the messenger RNA (mRNA) levels of the estrogen receptor (ER)-regulated genes progesterone receptor and steroid derived factor-1. Therefore, blocking estrogen signaling was prospective in targeted breast cancer therapy [102]. Another SK1 inhibitor, Ski (2-(p-hydroxyanilino)-4-(p-chlorophenyl)thiazole), induced the proteasomal degradation of SK1 in human androgen-sensitive LNCaP prostate cancer cells [103]. Further research is needed to illustrate the specific relationship between Ski inhibitors and hormone-mediated diseases. Cancer studies with SphK2 are less common than those with SphK1. Therefore, illustrating the possibility of SphK2 associated disease is difficult. In an early study, Van Brocklyn JR found that RNA interference of SphK2 expression inhibited glioblastoma cell proliferation more potently than did SphK1 knockdown [104]. One study found that in xenograft nude mice, SphK2-deficient tumors displayed a pronounced anti-tumor phenotype, including increased pro-inflammatory markers NO, TNF-alpha, and IL-12 [105]. A subsequent study proposed that ABC294640, a selective inhibitor of SphK1 that modulated the S1P level, could antagonize cancers such as breast cancer and ovarian cancer [106, 107]. The mechanism may be via the inhibition of tumor cell migration, concomitant with loss of microfilaments [98]. Correspondingly, we could develop clinical or intervention strategies to cure or ease disease.

FTY720, an immunosuppressive small molecule drug, bound to and eventually induced the internalization and degradation of one out of the five known S1P cell surface receptors, which prevented its function as a sensor of S1P gradients [108]. FTY720, which is phosphorylated in vivo by SphK2, could prevent T and B cell egress from lymph nodes (LN) and Peyer’s patches [109], as the S1P/S1PR axis is also important for B cell migration through the lymph node follicle [18]. FTY720, an agonist of S1P, has been approved for the treatment of multiple sclerosis. Its ligands were for all of the S1PRs, except S1PR2. The clinical efficacy of FTY720 has been attributed to its ability to promote the retention of naive T cells and central memory T cells (including autoreactive T_H_17 cells) in lymph nodes, preventing terminally differentiated effector T cells and effector memory T cells from entering the central nervous system and driving pathological responses. On the other hand, FTY720 minimally affected peripheral effector memory T cells, which were important for protection against infection [109–111].

## Conclusions

Immune microenvironment is a changeable zone in which large amounts of molecules, metabolites, and electrochemical substances weave mixed network. The function of sphingosine not only existed in its relation with other sterols in chemistry but it also causes huge biological influences through SphKs/S1P/S1PR1 axis, including physiological/pathological processes, e.g., hemopoietic system, lymphocytes internalization, allergic response, ischemia reperfusion injury, inflammatory bowel disease, and heterogeneity of carcinoma. In our retrospective study, many researchers illustrated SphKs/S1P/S1PR1’s functions via “inside-out signaling”, and some targeted drugs focused on SphKs/S1P/S1PR1 axis impact abovementioned physiological or pathological processes (like PI3K/Akt/NF-κB pathway). Although it is difficult to keep pace with the rapidly moving target of S1P in the immune system, the development of a second generation of drugs with improved specificity and efficacy will provide new treatment strategies for these inflammatory disorders and, moreover, will enhance our understanding of how this “simple” sphingolipid metabolite functions both the inside and outside of cells.
